# Derby County Asylum, Mickleover

**Published:** 1853-01-01

**Authors:** 


### DERBY COUNTY ASYLUM, MICKLEOVER.

No report of this asylum had been issued at the time of our going to press; but, from a personal visit, we are enabled to state as follows:—Two hundred and three patients have been admitted into the asylum since its opening, in August, 1851. Of these, one hundred and ten were males, and ninety-three were females. At the above date, the institution was in a very unfinished condition—having only one gallery on each side in a state to receive patients. At the end of the year 1851, eighty-two patients had been received. At our visit in September last, we found the wards well furnished, and all the internal arrangements very complete. A great deal of work was going forwards in the gardens and grounds, and the various walks and roads were receiving necessary attention. Many workmen were employed in erecting a wall around a large portion of the estate. The asylum is about five miles south-west of the Derby station, situated in a beautiful locality, commanding extensive prospects over the Trent valley, to the Leicester hills and Charnwood forest. This fine institution has been so fully described in Dr. Conolly's work on Lunatic Asylums, that we need not enter into details further than to state, that the galleries are remarkably cheerful, the airing-courts spacious and well constructed, and throughout the whole arrangements there is an entire absence of anything like imprisonment or gloom. At the time of our visit a very maniacal patient, admitted a few days previous, was in a parturient state, and exposed to great danger from the combined influence of mania and flooding. The recumbent position could not be permanently secured, without the exertion of several nurses. In consultation with Dr. Ramsay, who was superintending the asylum in the temporary absence of Dr. Hitchman, we prescribed chloroform; and are happy to hear that the patient did well.

Of the two hundred and three patients admitted, thirty-one only, uncomplicated by epilepsy, paralysis, or extreme old age, have been received within one month of their malady; of these, twenty-four have been discharged cured, and five of the others are under early treatment, with every prospect of a successful issue. This fact is one among many others, illustrative of the importance of sending patients from home for treatment, at the very earliest stage of the malady.

Fourteen patients have died in the above sixteen months; the deaths of twelve of the above patients were predicted to their friends on their admission, in consequence of their being greatly exhausted by general paralysis, old age, or other causes.

We found the patients as a body very healthful, cheerful, and contented, and employed in considerable numbers upon the farm and garden.

